# A 4-Week Intervention Involving Mobile-Based Daily 6-Minute Micro-Sessions of Functional High-Intensity Circuit Training Improves Strength and Quality of Life, but Not Cardio-Respiratory Fitness of Young Untrained Adults

**DOI:** 10.3389/fphys.2018.00423

**Published:** 2018-05-09

**Authors:** Billy Sperlich, Lea-Sofie Hahn, Antonia Edel, Tino Behr, Julian Helmprobst, Robert Leppich, Birgit Wallmann-Sperlich, Hans-Christer Holmberg

**Affiliations:** ^1^Integrative and Experimental Exercise Science and Training, Institute for Sport Sciences, University of Würzburg, Würzburg, Germany; ^2^Institute for Sport Sciences, University of Würzburg, Würzburg, Germany; ^3^The Swedish Winter Sports Research Centre, Mid Sweden University, Östersund, Sweden; ^4^School of Kinesiology, The University of British Columbia, Vancouver, BC, Canada; ^5^School of Sport Sciences, UiT The Arctic University of Norway, Tromsø, Norway

**Keywords:** aerobic fitness, body composition, functional training, mHealth, power training, V800, wearable, Web-based apps

## Abstract

The present study was designed to assess the psycho-physiological responses of physically untrained individuals to mobile-based multi-stimulating, circuit-like, multiple-joint conditioning (Circuit_HIIT_) performed either once (1xCircuit_HIIT_) or twice (2xCircuit_HIIT_) daily for 4 weeks. In this single-center, two-arm randomized, controlled study, 24 men and women (age: 25 ± 5 years) first received no training instructions for 4 weeks and then performed 4 weeks of either 1xCircuit_HIIT_ or 2xCircuit_HIIT_ (5 men and 7 women in each group) daily. The 1xCircuit_HIIT_ and 2xCircuit_HIIT_ participants carried out 90.7 and 85.7% of all planned training sessions, respectively, with average heart rates during the 6-min sessions of 74.3 and 70.8% of maximal heart rate. Body, fat and fat-free mass, and metabolic rate at rest did not differ between the groups or between time-points of measurement. Heart rate while running at 6 km⋅h^-1^ declined after the intervention in both groups. Submaximal and peak oxygen uptake, the respiratory exchange ratio and heart rate recovery were not altered by either intervention. The maximal numbers of push-ups, leg-levers, burpees, 45°-one-legged squats and 30-s skipping, as well as perception of general health improved in both groups. Our 1xCircuit_HIIT_ or 2xCircuit_HIIT_ interventions improved certain parameters of functional strength and certain dimensions of quality of life in young untrained individuals. However, they were not sufficient to enhance cardio-respiratory fitness, in particular peak oxygen uptake.

## Introduction

Low levels of cardiorespiratory fitness (CRF) and physical activity (PA) are two major independent risk factors for both cardiovascular disease (CVD) and all-cause mortality (Blair et al., 1989; DeFina et al., 2015). Programs designed to improve cardiovascular, metabolic and psychological health involving repeated short-to-long bouts of high-intensity exercise with intervals of recovery (referred to as high-intensity interval training or HIIT) have begun to replace those based on low-intensity high-volume exercise (Kessler et al., 2012; Elliott et al., 2014; Little and Francois, 2014; Gielen et al., 2015; Schmitt et al., 2016). In addition to encompassing an unlimited number of protocols with different work-to-rest ratios, orders of loading and distributions of training intensity, HIIT requires less time and perceived lack of time appears to be a major reason for not exercising (Godin et al., 1994).

Accumulating evidence indicates that low-volume HIIT (e.g., 4–6 30-s cycle sprints separated by ∼4 min of recovery or 10 60-s work bouts at a constant intensity that elicits ∼90% of maximal heart rate, separated by 60 s of recovery) induces physiological remodeling in several ways that improve metabolic control in skeletal muscle, as well as cardiovascular function (Gibala et al., 2012). Such metabolic improvement is beneficial to numerous individuals, including previously sedentary, overweight individuals in whom insulin sensitivity can be improved by this type of exercise (Whyte et al., 2010).

As has shown to be the case for traditional endurance-based HIIT, the relatively novel functional training/fitness involving multi-stimulating, circuit-like, multiple-joint, high-intensity exercise (Circuit_HIIT_) improves body composition, as well as cardiovascular and functional fitness and certain aspects of quality of life (Sperlich et al., 2017). Improved CRF (e.g., maximal oxygen uptake) and muscular strength are associated with improved dimensions of health (Nikander et al., 2010; Thomas et al., 2017) and fewer premature deaths (Bouchard et al., 2015). However, although time-saving bouts of sprint-based HIIT enhance certain dimensions of health, this type of training is extremely demanding; may not be safe, tolerable or appealing for certain individuals (Gibala et al., 2012); and may not improve whole-body strength.

Mobile web-based technology is growing rapidly and now offers more than 100,000 applications (Modave et al., 2015) designed to improve various aspects of health and physical fitness (Sama et al., 2014; Modave et al., 2015). Digital technology may effectively reduce the length of contact time between individuals seeking care and health or exercise professionals (Atkinson and Gold, 2002) and thereby the costs involved. Numerous mobile apps presently available, especially in iTunes and Google Play, support a variety of Circuit_HIIT_-like exercises lasting 5–8 min, micro-sessions which may, if performed for approximately 75 min each week (i.e., approximately twice a day), be in line with current recommendations for improving and maintaining physical fitness and health (Garber et al., 2011).

Improvement in neuromuscular function through aerobic exercise is associated with other health benefits, including reduced lower-back pain (Cortell-Tormo et al., 2018), enhanced bone density (Petersen et al., 2017), better body composition (Cholewa et al., 2017) and more favorable psychological perception (e.g., self-satisfaction, self-esteem and body image) (Sothern et al., 1999). In this context, short-term (i.e., 4-week) resistance training has been proven to improve the one-repetition concentric strength of sedentary, healthy men by 19% (Raue et al., 2005), as well as to elevate the maximal isometric torque of both healthy men and women (Mayhew et al., 1995).

Micro-sessions of Circuit_HIIT_ may offer one approach to circumventing lack of time as a reason for not exercising (Godin et al., 1994). However, a recent systematic review highlighted the fact that physical activity promoted by smart phones leads to heterogeneous outcomes (Bort-Roig et al., 2014) and does not apply evidence-based guidelines to aerobic and resistance training (Knight et al., 2015). Thus, although promoted extensively online, surprisingly little scientific evaluation of the effect of web-based interventions, including Circuit_HIIT_, on cardiorespiratory and metabolic functions, body composition, functional strength and quality of life of untrained individuals is presently available.

Therefore, we have compared here the psycho-physiological responses of physically untrained individuals to a web-based multi-stimulating, circuit-like, multiple-joint conditioning program (Circuit_HIIT_) performed either once (1xCircuit_HIIT_) or twice (2xCircuit_HIIT_) daily for 4 weeks.

## Materials and Methods

### Participants

For this single-center, two-arm randomized, controlled study, 24 men and women (major baseline characteristics are summarized in **Table 1**) were recruited via social media and bulletins. They were assigned randomly to perform a 6-min web-based micro-session of functional high-intensity circuit training, individually designed on the basis of their peak oxygen uptake at baseline, either once (1xCircuit_HIIT_) or twice (2xCircuit_HIIT_) (5 men and 7 women in each case) daily for 4 weeks. *T*-test analysis of VO_2*peak*_ prior to the intervention demonstrated no difference in this respect between the two groups (*p* = 0.75; *T* = 0.32).

**Table 1:** Anthropometric characteristics of and peak oxygen uptake by our participants at baseline.

Circuit_HIIT_ group	Sex	Height [cm]	Mass [kg]	Body mass index [kg⋅m^-2^]	VO_2*max*_ [ml⋅min^-z1^⋅kg^-1^]
1x	Men	190.8 6.3	85.2 2.1	23.5 ± 1.5	45.7 ± 1.1
	Women	169.6 4.1	67.0 7.0	23.3 ± 2.2	38.5 ± 5.1
	Combined	176.7 11.2	73.1 10.6	23.3 ± 1.9	40.9 ± 5.5
2x	Men	180.0 8.1	83.2 13.7	25.6 ± 3.5	40.4 ± 5.1
	Women	167.86 3.7	62.6 9.6	22.2 ± 3.1	39.8 ± 8.5
	Combined	172.92 8.4	71.2 15.2	23.6 ± 3.6	40.1 ± 7.0


All were informed in detail about the design of the study, including the potential risks and benefits, before providing their written consent to participate. The inclusion criteria were an age of 18–40 years; lack of any frequent participation in endurance or strength exercise programs for at least 6 months prior to the study; no daily intake of medication; and for inclusion in the analysis, completion of at least 80% of the training sessions.

All procedures were conducted in accordance with the Declaration of Helsinki and the experimental protocol was approved by the ethical review board of the Sport Science Institute of the University of Würzburg.

#### Overall Study Design

**FIGURE 1:**
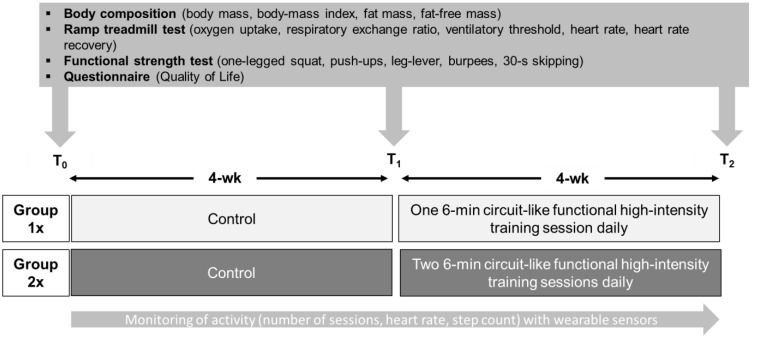
The study design, including testing prior to (T_0_) and after (T_1_) the baseline period and following the intervention (T_2_).

Two to 3 days prior to (T_0_) and 2 days after the 4-week baseline period (T_1_), as well as two to 3 days following the 4-week Circuit_HIIT_ intervention (T_2_), all underwent assessment of body composition, a treadmill ramp test designed to assess cardiorespiratory and metabolic variables, and numerous tests of functional movement and strength, as well as filling in a questionnaire concerning quality of life (**Figure 1**).

### The Interventions

Each of the 4 weeks of intervention involved a different 6-min micro-session of Circuit_HIIT_. After each week, each participant received a web-based video (designed for use either on a desktop computer or smartphone/tablet) of an instructor carrying out functional Circuit_HIIT_ movements [for details, see (www.sportsandscience.de, 2017a,b,c,d)] and were instructed to follow these movements with high-intensity, with monitoring of this intensity by heart rate. The 2xCircuit_HIIT_ group was instructed to recover for 2–3 h between the two sessions on the same day. All sessions and other free-time activities were monitored by a multi-sensor device worn on the wrist (Polar V800, Polar Electro Oy, Kempele, Finland).

#### Anthropometric Data and Body Composition

Height while standing barefoot was measured with a folding yardstick. Body, fat and fat-free mass, as well as metabolic rate at rest were assessed with a four-electrode bio-impedance scale (Model 1609N; Tanita Corp., Tokyo, Japan) and the body-mass-index (BMI; in kg⋅m^-2^) then calculated. Since dehydration may affect such bio-impedance analysis, all participants were instructed to drink 500 mL of water 1 h before these measurements.

#### The Treadmill Test

After running at 6 km⋅h^-1^ for 5 min on a treadmill (Mercury, h/p/Cosmos Sports & Medical GmbH, Nußdorf, Germany), submaximal heart rate, oxygen uptake and the respiratory exchange ratio were all evaluated. Thereafter, for determination of the peak values of these same parameters, the speed was increased by 1 km⋅h^-1^ each minute until exhaustion. As recommended earlier (Poole and Jones, 2017), a ramp test involving constant work at approximately 110% VO_2peak_ was performed shortly thereafter for validation and the higher of the two values obtained considered to be VO_2peak_. Heart rate recovery (HR_recovery_) was defined as the reduction in heart rate while standing still during the first 60 s after termination of the ramp test.

Throughout the testing, oxygen uptake, the respiratory exchange ratio and heart rate were monitored with an open circuit breath-by-breath gas and volume analyzer (Metamax 3B, Cortex Biophysik GmbH, Leipzig, Germany), employing standard algorithms to compensate for the delay between oxygen consumption and generation of the signal. All respiratory data and heart rates were averaged over 30-s intervals. The oxygen uptake at 6 km⋅h^-1^ was considered to be an indicator of running economy, as described elsewhere (Barnes and Kilding, 2015). Post-exercise heart rate recovery is considered to be a marker for general hemodynamic adjustments and this 60-s recovery is correlated to changes in training status (Daanen et al., 2012).

#### Functional Strength and 30-s Skipping

To test functional strength, all participants were asked to perform as many push-ups, leg-levers, burpees and 45°-one-legged squats in 1 min as they could (separated by 3 min of recovery). All of this functional testing was adopted from a previous study (Sperlich et al., 2017). Repeated testing on two different occasions (3 days apart; n = 10) revealed a good test-retest reliability based on the intraclass correlation coefficient (ranging from 0.93 to 0.98) and technical error measurement (ranging from 2.8 to 4.8%) results.

When performing the 45°-one-legged squat, all participants began in an upright position with both knees stretched (180°) and both hands on the waist with an elbow angle of approximately 90°. The participant was instructed to bend the knee of the leg he/she was standing on to 45° and avoid ground contact with the other foot. (A larger knee angle was not possible, since our untrained participants were not strong or stable enough). After bending, the participant returned to the upright position with both knees stretched (180°), thereby ending the cycle. This testing started with the right leg; after a 30-s break, the left leg was tested in the same manner and the average number of repetitions with both legs used for statistical analysis.

The push-ups at T_0_, T_1_ and T_2_ were performed in the same manner. When the participant was unable to raise his/her elongated torso and lower-body from the floor, he/she was allowed to perform subsequent push-ups while kneeling. One push-up began when the torso was lifted from the floor and ended when it touched the floor again.

During the leg lever test the participant lay stretched out on a fitness mat with both arms and hands held close in to the body. The cycle began with both legs, knees fully extended, being raised simultaneously to an angle of 60° at the hip. The cycle ended when both legs were lowered slightly without the heels touching the ground. Thereafter, this cycle was repeated, and the number of repetitions employed for statistical analysis.

A valid burpee began in the standing position, from which the participant moved into a squatting position with the two legs simultaneously kicking back, to land in a plank position on both hands and tiptoes. Then, the participant returned to the squat position and ended the burpee with a vertical jump. This cycle was repeated, and the number of repetitions employed for statistical analysis.

Skipping performance was assessed as the maximal number of skips during a 30-s period, as recorded by the OptoJump apparatus (MicroGate Srl, Bolzano, Italy).

### Assessment of Quality of Life

At T_0_, T_1,_ and T_2_, all completed the German version of the health-related quality of life questionnaire (SF-36), which has been confirmed to be both valid and reliable (Bullinger et al., 1995). This questionnaire assesses general and mental health, physical and social functioning, vitality, bodily pain, and the impact of physical and emotional limitations, with higher scores (0–100) reflecting better quality of life. The questionnaire was distributed to the participants by a member of our research group prior to physiological testing and later collected by the same person.

### Monitoring Activity and Training

Collection of data on activity was initiated immediately after the first ramp test (T_0_) and terminated after the last ramp test approximately 8 weeks later (T_2_). Each individual wore a multisensory device (Polar V800, Polar Electro Oy, Kempele, Finland) on the wrist on which they usually wore a watch for the entire study period (with the exception of short intervals for charging). When the participants were not exercising, the Polar V800 instrument without a chest-belt recorded the number of steps and intensity of daily activities automatically, with a validity comparable to that of accelerometers (Hernandez-Vicente et al., 2016). During each session of Circuit_HIIT_ they wore a chest-belt and activated monitoring of heart rate manually. Each participant uploaded all these data for web-based storage via the Polar Flow software. This software provides the number of steps taken daily, the number of waking hours spent sedentary each day [<1.5 metabolic equivalents (MET)], and the time spent performing light (1.5–3 MET), moderate (3–6 MET) and vigorous physical activity (>6 MET) each day. For the analysis of each Circuit_HIIT_ session, we averaged the heart rate data from the last 2 min.

### Statistical Analyses

When the distribution of the data was examined with the Shapiro–Wilk test, this distribution was found to be non-normal in the case of physical activity and health-related quality of life (SF-36). Since further data transformation did not improve this situation, we continued with parametric testing. Repeated measures ANOVAs (time points: baseline and intervention for physical activity and T_0_, T_1_, T_2_ for all other variables) were performed with the physical activity, cardiorespiratory and metabolic parameters, body composition, functional strength and quality of life as the intra-subject factors and group (1xCircuit_HIIT_ or 2xCircuit_HIIT_) as the inter-subject factor. An alpha of ρ < 0.05 with Bonferroni adjustment for multiple comparisons was considered significant. In addition, the values obtained were evaluated by calculating the effect size partial eta-square (η*_p_*^2^). The means and standard deviations (SD) of all data sets were calculated and all statistical tests carried out in the SPSS 22.0 software package for Microsoft (SPSS Inc., Chicago, IL, United States).

## Results

### Activity Measurements (**Table 2**)

For the 1xCircuit_HIIT_ group, valid data (i.e., >480 min/day) were obtained for 19.2 ± 1.5 weekdays and 7.4 ± 1.2 weekend days during the 4-week baseline period and 18.8 ± 1.8 weekdays and 6.9 ± 1.4 weekend days during the 4-week intervention. The corresponding values for the 2xCircuit_HIIT_ group were 18.5 ± 2.9 and 7.6 ± 1.2 vs. 18.9 ± 1.9 and 7.4 ± 1.3 days, respectively. The mean sedentary time and duration of light, moderate and vigorous physical activity are presented in **Table 2**.

**Table 2:** Activity parameters (means ± SD) for the 1xCircuit_HIIT_ and 2xCircuit_HIIT_ groups during the baseline (first 4-week) and intervention (second 4-week) periods.

Parameter	Days	Circuit_HIIT_ group	Baseline	Intervention	*p*(T)/ *p*(G)/ *p*(T × G)	$\eta_{p}^{2}$ (T)/ $\eta_{p}^{2}$ (G)/ $\eta_{p}^{2}$ (T × G)	*F(T)/ F*(G)/ *F*(T × G)
Days for which valid	Weekdays	1x	19.2 ± 1.5	18.8 ± 1.8	0.962	0.000	0.002
data were obtained [*n*]		2x	18.5 ± 2.9	18.9 ± 1.9	0.727	0.006	0.125
					0.491	0.023	0.491
	Saturday–Sunday	1x	7.4 ± 1.2	6.9 ± 1.4	0.275	0.057	1.259
		2x	7.6 ± 1.2	7.4 ± 1.3	0.424	0.031	0.664
					0.745	0.005	0.109
Total time during which	Weekdays	1x	20.8 ± 3.8	20.1 ± 4.0	0.466	0.026	0.551
the sensor was worn [h]		2x	21.1 ± 4.7	20.9 ± 3.5	0.731	0.006	0.121
					0.691	0.008	0.163
	Saturday–Sunday	1x	19.3 ± 5.2	19.6 ± 4.7	0.689	0.008	0.165
		2x	21.4 ± 4.1	20.4 ± 5.2	0.440	0.029	0.620
					0.486	0.023	0.504
Steps [*n*]	Weekdays	1x	9788 ± 2773	9183 ± 2339	0.790	0.004	0.073
		2x	10142 ± 4496	10527 ± 3965	0.553	0.018	0.365
					0.240	0.068	1.465
	Saturday–Sunday	1x	8958 ± 2507	9020 ± 2469	0.738	0.006	0.115
		2x	8795 ± 4972	9177 ± 4972	0.999	0.000	0.000
					0.809	0.003	0.069
Sedentary time (<1.5 MET)	Weekdays	1x	9.3 ± 1.9	9.3 ± 2.4	0.616	0.014	0.260
during waking hours [h]		2x	8.9 ± 2.1	9.2 ± 1.7	0.755	0.005	0.100
					0.651	0.011	0.212
	Saturday–Sunday	1x	8.6 ± 2.7	8.0 ± 2.8	0.141	0.111	2.361
		2x	9.0 ± 1.7	8.0 ± 2.8	0.835	0.002	0.045
					0.788	0.004	0.075
Light physical activity	Weekdays	1x	4.1 ± 1.0	3.9 ± 1.1	0.341	0.043	0.948
(1.5–3 MET) [h]		2x	4.7 ± 1.8	4.3 ± 1.8	0.351	0.042	0.911
					0.610	0.013	0.268
	Saturday–Sunday	1x	4.0 ± 0.9	4.2 ± 1.3	0.676	0.009	0.180
		2x	4.6 ± 2.1	4.0 ± 2.1	0.747	0.005	0.107
					0.341	0.043	0.948
Moderate physical activity	Weekdays	1x	0.7 ± 0.2	0.6 ± 0.2	0.415	0.032	0.690
(3–6 MET) [h]		2x	0.8 ± 0.3	0.7 ± 0.3	0.494	0.023	0.485
					0.913	0.001	0.012
	Saturday–Sunday	1x	0.6 ± 0.2	0.5 ± 0.2	0.340	0.046	0.957
		2x	0.6 ± 0.4	0.6 ± 0.3	0.717	0.007	0.135
					0.340	0.046	0.957
Vigorous physical activity	Weekdays	1x	0.2 ± 0.1	0.2 ± 0.1	0.024	0.219	5.880
(>6 MET) [h]		2x	0.2 ± 0.1	0.3 ± 0.2	0.621	0.012	0.253
					0.211	0.073	1.664
	Saturday–Sunday	1x	0.1 ± 0.1	0.2 ± 0.2	0.029	0.206	5.456
		2x	0.1 ± 0.1	0.2 ± 0.3	0.800	0.003	0.066
					0.395	0.035	0.753


**p, probability; $\eta_{p}^{2}$, effect size partial eta-square; MET,metabolic equivalent; T, global effect of time; G, global effect of Group; T × G, global effect of Time × Group.**

The only difference between the intervention and baseline periods was that both groups performed more vigorous activity on all days of the week during the intervention.

### Training Adherence and Heart Rate

During the intervention, participants in the 1xCircuit_HIIT_ and 2xCircuit_HIIT_ groups performed 90.7 and 85.7%, respectively, of all the planned training sessions, achieving 74.3 and 70.8% of maximal heart rate during these micro-sessions.

### Pre–Post Testing

All of the parameters measured at T_0_, T_1,_ and T_2_ with accompanying statistical analyses are presented in **Tables 2**–**5**. Body, fat and fat-free mass and metabolic rate at rest were the same for both groups and at all of these time-points (**Table 3**).

**Table 3:** Anthropometric parameters (means ± SD) for the 1xCircuit_HIIT_ and 2xCircuit_HIIT_ groups before (T_0_) and after (T_1_) the 4-week baseline period and following the 4-week intervention (T_2_).

Parameter	Circuit_HIIT_ group	T_0_	T_1_	T_2_	*p*(T)/ *p*(G)/ *p*(T × G)	$\eta_{p}^{2}$ (T)/ $\eta_{p}^{2}$ (G)/ $\eta_{p}^{2}$ (T × G)	*F(T)/ F*(G)/ *F*(T × G)
Body mass [kg]	1x	73.1 ± 10.6	73.2 ± 10.7	73.6 ± 11.1	0.462	0.071	0.801
	2x	71.2 ± 15.2	71.5 ± 15.2	71.4 ± 15.1	0.720	0.006	0.132
					0.317	0.104	1.213
Fat-free mass [%]	1x	33.3 ± 4.7	32.9 ± 4.5	33.2 ± 5.2	0.119	0.184	2.361
	2 x	34.6 ± 5.6	34.0 ± 6.0	34.6 ± 5.9	0.560	0.016	0.351
					0.868	0.013	0.143
Fat mass [kg]	1 x	26.5 ± 7.0	27.2 ± 6.7	27.1 ± 7.2	0.138	0.172	2.182
	2 x	24.8 ± 8.2	25.7 ± 8.9	24.8 ± 8.9	0.565	0.015	0.341
					0.725	0.030	0.327
Metabolic rate at rest [kcal]	1x	1551 ± 208	1548 ± 211	1555 ± 242	0.815	0.019	0.207
	2x	1555 ± 269	1557 ± 268	1560 ± 265	0.952	0.000	0.004
					0.629	0.043	0.473


**p, probability; $\eta_{p}^{2}$, effect size partial eta-square; T, global effect of time; G, global effect of Group; T × G, global effect of Time × Group.**

Heart rate while running at 6 km/h declined with time (*p* = 0.017) in both groups. All other variables (i.e., submaximal oxygen uptake and the respiratory exchange ratio, as well as peak oxygen uptake and maximal heart rate) were unaltered (**Table 4**).

**Table 4:** Cardio-respiratory and metabolic parameters (means ± SD) for the 1xCircuit_HIIT_ and 2xCircuit_HIIT_ groups before (T_0_) and after (T_1_) the 4-week baseline period and following the 4-week intervention (T_2_).

Parameter	Circuit_HIIT_ group	T_0_	T_1_	T_2_	*p*(T)/ *p*(G)/ *p*(T × G)	*$\eta_{p}^{2}$* (T)/ *$\eta_{p}^{2}$* (G)/ *$\eta_{p}^{2}$* (T × G)	*F(T)/ F*(G)/ *F*(T × G)
*At submaximal running speed*							
Heart rate [bpm]	1x	139 ± 13	134 ± 17	133 ± 15	0.017^a,b^	0.336	5.054
	2x	136 ± 12	129 ± 12	129 ± 14	0.412	0.032	0.700
					0.871	0.014	0.139
Oxygen uptake [ml⋅min^-1^ kg^-1^]	1x	17.7 ± 1.3	17.2 ± 1.4	17.3 ± 1.3	0.458	0.072	0.811
	2x	17.6 ± 2.5	17.1 ± 1.5	17.1 ± .8	0.869	0.001	0.028
					0.952	0.005	0.049
Respiratory exchange ratio	1x	0.81 ± .02	0.82 ± .03	0.81 ± .04	0.495	0.065	0.728
	2x	0.81 ± .05	0.82 ± .04	0.81 ± .04	0.742	0.005	0.111
					0.889	0.011	0.118
*At the point of exhaustion*							
Maximal heart rate [bpm]	1x	192 ± 8	191 ± 8	191 ± 8	0.791	0.022	0.237
	2x	189 ± 10	188 ± 9	188 ± 9	0.365	0.037	0.856
					0.951	0.005	0.050
Maximal oxygen uptake [ml⋅min^-1^]	1x	40.9 ± 5.5	41.2 ± 6.5	40.7 ± 6.9	0.286	0.112	1.328
	2x	40.1 ± 7.0	41.3 ± 7.3	41.7 ± 6.3	0.977	0.000	0.001
					0.476	0.068	0.770
*After the point of exhaustion*							
Heart rate recovery [bpm]	1x	28.8 ± 12.1	31.1 ± 12.2	32.7 ± 10.7	0.119	0.192	2.373
	2x	28.6 ± 8.9	33.3 ± 8.9	26.9 ± 7.6	0.742	0.005	0.111
					0.143	0.177	2.143


**p, probability; $\eta_{p}^{2}$, effect size partial eta-square; T, global effect of time; G, global effect of Group; T × G, global effect of Time × Group; a, T_0_ vs. T_1_ (p < 0.05); b, T_0_ vs. T_2_ (p < 0.05).**

The maximal numbers of push-ups, leg-levers, burpees, 45°-one-legged squats and 30-s skipping was greater at T_2_ than T_1_ for the participants in both groups, with no differences between 1xCircuit_HIIT_ and 2xCircuit_HIIT_ (**Table 5**).

**Table 5:** Tests of funtional strength (means ± SD) for the 1xCircuit_HIIT_ and 2xCircuit_HIIT_ groups before (T_0_) and after (T_1_) the 4-week baseline period and following the 4-week intervention (T_2_).

Test [*n*]	Circuit_HIIT_ group	T_0_	T_1_	T_2_	*p*(T)/ *p*(G)/ *p*(T × G)	*$\eta_{p}^{2}$*(T)/*$\eta_{p}^{2}$*(G)/ *$\eta_{p}^{2}$*(T × G)	*F(T)/F*(G)/ *F*(T × G)
Push-ups	1x	28.9 ± 13.4	32.9 ± 12.9	35.6 ± 8.5	0.001^b,c^	0.520	10.838
	2x	27.8 ± 9.9	28.6 ± 9.0	36.3 ± 13.1	0.719	0.006	0.133
					0.109	0.199	2.486
Leg-levers	1x	24.3 ± 7.2	24.3 ± 6.5	26.2 ± 7.3	0.001^b,c^	0.514	11.093
	2x	22.7 ± 5.3	23.1 ± 5.9	27.4 ± 6.4	0.827	0.002	0.049
					0.173	0.154	1.909
Burpees	1x	16.9 ± 3.2	16.5 ± 2.8	19.3 ± 3.0	<0.001^b,c^	0.572	13.365
	2x	16.4 ± 4.7	17.4 ± 4.4	20.7 ± 6.4	0.719	0.006	0.133
					0.059	0.246	3.266
45°-one-legged squats	1x	28.7 ± 9.7	35.3 ± 11.1	42.7 ± 14.4	<0.001^a,b,c^	0.699	24.389
	2x	30.8 ± 10.9	36.6 ± 13.7	42.5 ± 12.9	0.818	0.002	0.054
					0.832	0.017	0.186
30-s Skipping	1x	104.8 ± 22.6	101.8 ± 17.1	115.4 ± 31.7	0.015^b,c^	0.332	5.211
	2x	87.6 ± 23.9	92.4 ± 18.5	106.5 ± 25.3	0.158	0.089	2.138
					0.600	0.047	0.523


**p, probability; $\eta_{p}^{2}$, effect size partial eta-square; T, global effect of time; G, global effect of Group; T × G, global effect of Time × Group; a, T_0_ vs. T_1_ (p < 0.05); b, T_0_ vs. T_2_ (p < 0.05); c, T_1_ vs. T_2_ (p < 0.05).**

Perception of general health improved following both interventions (**Table 6**).

**Table 6:** Quality of life (SF36; arbitrary units, means ± SD) for the 1xCircuit_HIIT_ and 2xCircuit_HIIT_ groups before (T_0_) and after (T_1_) the 4-week baseline period and following the 4-week intervention (T_2_).

Item	Circuit_HIIT_ group	T_0_	T_1_	T_2_	*p*(T)/*p*(G)/ *p*(T × G)	*$\eta_{p}^{2}$*(T)/*$\eta_{p}^{2}$*(G)/ *$\eta_{p}^{2}$*(T × G)	*F(T)/ F*(G)/ *F*(T × G)
Physical functioning	1x	95.5 ± 7.6	94.5 ± 9.1	94.5 ± 8.8	0.964	0.004	0.037
	2x	98.6 ± 2.3	98.6 ± 2.3	99.1 ± 3.0	0.038	0.197	4.913
					0.945	0.006	0.057
Impact of physical limitations	1x	88.6 ± 20.5	79.5 ± 40.0	72.7 ± 37.8	0.403	0.091	0.954
	2x	95.5 ± 10.1	81.8 ± 33.7	97.7 ± 7.5	0.153	0.100	2.210
					0.323	0.112	1.199
Pain	1x	84.9 ± 19.1	77.4 ± 22.6	76.9 ± 19.4	0.658	0.043	0.428
	2x	87.5 ± 16.0	87.8 ± 11.6	88.2 ± 16.8	0.188	0.085	1.856
					0.598	0.053	0.528
Perception of general health	1x	74.8 ± 16.6	75.0 ± 12.7	78.3 ± 12.1	0.043^a^	0.282	3.735
	2x	76.8 ± 14.0	81.3 ± 14.6	83.3 ± 13.7	0.434	0.031	0.636
					0.622	0.049	0.487
Vitality	1x	60.5 ± 11.3	58.2 ± 15.2	57.7 ± 13.8	0.206	0.153	1.717
	2x	60.9 ± 9.4	53.2 ± 18.2	55.9 ± 19.5	0.676	0.009	0.180
					0.668	0.042	0.412
Social functioning	1x	89.8 ± 15.6	89.8 ± 16.6	95.5 ± 10.1	0.722	0.034	0.331
	2x	86.4 ± 22.7	81.8 ± 22.6	84.1 ± 23.1	0.196	0.082	1.786
					0.767	0.028	0.269
Impact of emotional limitations	1x	90.9 ± 21.6	87.9 ± 27.0	90.9 ± 15.6	0.832	0.019	0.186
	2x	75.8 ± 33.6	69.7 ± 45.8	69.7 ± 40.7	0.162	0.113	2.547
					0.893	0.012	0.114
Mental health	1x	78.2 ± 8.1	78.9 ± 10.7	80.4 ± 9.5	0.613	0.050	0.501
	2x	78.9 ± 10.6	73.7 ± 15.2	73.8 ± 14.1	0.391	0.037	0.770
					0.191	0.160	1.810


**p, probability; $\eta_{p}^{2}$, effect size partial eta-square; T, global effect of time; G, global effect of Group; T × G, global effect of Time × Group; a, T_0_ vs. T_2_ (p < 0.05).**

## Discussion

Our major novel findings here concerning the psycho-physiological responses of young untrained individuals to 4 weeks of either 1xCircuit_HIIT_ or 2xCircuit_HIIT_ were as follows: (1) Neither intervention influenced the number of steps taken daily or amount of sedentary time or light or moderate physical activity, but the amount of vigorous physical activity by both groups was higher during the intervention than baseline period. (2) Body composition remained unchanged. (3) With the exception of heart rate while running at 6 km/h, no cardiorespiratory or metabolic parameters differed between the time-points of measurements or between the groups. (4) All parameters related to functional strength were improved following both interventions. And, finally, (5) perception of general health was also better after both 1xCircuit_HIIT_ and 2xCircuit_HIIT_.

In the current investigation adherence to the prescribed exercise program was quite high (>85% of all planned sessions), despite the fact that there was no social networking by our participants nor did we provide reminders via social media, a calendar for scheduling or encouragement when lapses occurred – all effective strategies in connection with mobile apps designed for weight loss (Pagoto et al., 2013). This high degree of adherence may reflect the fact that the intervention was relatively short, scientific, and detailed, as well as the fact that the individual sessions did not take much time. In this regards it was not unexpected that there was no change in the sedentary time or amounts of light and moderate physical activity.

Overall, the changes in functional strength were more pronounced than those in cardiorespiratory parameters. Although there is now a generally accepted definition of functional strength, we describe the training program employed here more specifically as multi-joint exercise designed to improve certain dimensions of strength and mobility in connection with everyday life, as well as the performance of specific sports. Depending on its intensity, duration and frequency, this type of strength training promotes the same changes in neuro-muscular structure and function as traditional strength training – i.e., release of inhibitory mechanisms, as well as improvements in intra- and intermuscular coordination (synchronization, recruitment and the rate coding of muscle fibers) and hypertrophic responses.

Although the hypertrophic response to eccentric and concentric signaling occurs immediately (Franchi et al., 2017), the gains in muscle protein mass may take several weeks (Moritani and deVries, 1979) or months (Zinner et al., 2017), depending on factors that include both training history and gender. Therefore, the improvement in functional strength caused by the present 4-week intervention (7.8–20.9 and 15.2–26.9% improvement with 1xCircuit_HIIT_ and 2xCircuit_HIIT_, respectively) probably reflects neural adaptation. Indeed, functional resistance training improves, e.g., health-related quality of life and physical fitness in women with chronic low-back pain (Cortell-Tormo et al., 2018) and it would be desirable to investigate the potential beneficial effects of our Circuit_HIIT_ in this context.

Interestingly, even though one group trained twice as much as the other, the parameters examined differed between them to only a small extent. Accordingly, from a practical point of view, a single 6-min daily session of functional Circuit_HIIT_ as described here appears to improve functional strength by 7.8–20.9%.

In contrast to these gains in functional strength, no cardiorespiratory changes were evoked here by either 1xCircuit_HIIT_ or 2xCircuit_HIIT_. Peak oxygen uptake, which limits ATP production via oxidative phosphorylation, is regarded as an integrative indicator of CRF and is closely associated with general health and premature death (Bassett and Howley, 2000; Bouchard et al., 2015). There may be several reasons why this important variable was unaltered by our interventions: First, these Circuit_HIIT_ interventions consisted primarily of resistance training (i.e., squats, lunges, push-ups, etc.). Second, meta-analyses recommend that in order to improve VO_2_peak, a program of resistance circuit-based training should involve 14–30 sessions over a period of 6–12 weeks, with each session lasting at least 20–30 min at an exercise intensity of 60–90% of the one-repetition maximum (Munoz-Martinez et al., 2017). Thus, a recent intervention involving similar functional movements, but longer sessions (>60 min vs. 6 min) over a longer period (9 vs. 4 weeks) than ours enhanced VO_2_peak approximately 10% (Sperlich et al., 2017). Apparently, our intervention did not induce any central cardiovascular adaptations because both the individual sessions and overall length were too short and the overall intensities of 74.3 and 70.8% of peak heart rate during 1xCircuit_HIIT_ and 2xCircuit_HIIT_, respectively, were not sufficiently high (i.e., approximately 90% of maximal heart rate).

This probably also explains the lack of any alteration in metabolism in response to our interventions. A reduction in the respiratory exchange ratio following a period of exercise is indicative of elevated lipid oxidation resulting from more extensive uptake of free fatty acids by working muscle, more efficient beta-oxidation, and down-regulation of glycolytic pathways (Dudley et al., 1987; Jansson and Kaijser, 1987; Brooks, 1997; Bergman and Brooks, 1999). Although endurance exercise increases fatty acid oxidation to a greater extent (Goodpaster et al., 2003; Berggren et al., 2004), even exercise of low-to-moderate intensity [i.e., ranging from 33 to 65% VO_2max_ (Achten et al., 2002)] also has such a beneficial effect (Jones et al., 1980; Broeder et al., 1991; Romijn et al., 1993; Bergman and Brooks, 1999). The heart rates during our Circuit_HIIT_ indicate that the mean intensity of this training (although not targeted) can be classified as “moderate.” Nevertheless, no changes in lipid or carbohydrate metabolism occurred, perhaps, once again, because the individual sessions and overall length of our interventions were too short.

Interestingly, 30-s skipping, which involves anaerobic metabolism (Quirk and Sinning, 1982), improved in both groups by 13.8–15.8%. Since rapid knee lifting is related to leg strength and neuro-muscular control (Trecroci et al., 2015), this improvement may reflect the gains in functional leg strength discussed above.

Our Circuit_HIIT_ participants performed primarily resistance training (i.e., squats, lunges, push-ups, etc.) with very little rest. Therefore, it is not surprising that their functional strength increased. However, the magnitude of this increase (as much as 48% for certain parameters) with only 6-min micro-sessions of Circuit_HIIT_ daily is remarkable.

Women who performed approximately 60 min of Circuit_HIIT_ each week experienced more pain after the 9-week intervention (Sperlich et al., 2017). Indeed, high-intensity strength training with eccentric components such as those involved in multi-stimulating, circuit-like, multiple-joint training induces muscle soreness. Although we did not assess pain during each training session, our participants perceived no more pain after the 1x and 2xCircuit_HIIT_ than during the baseline period. Furthermore, although the rate of injury associated with multi-stimulating, circuit-like, multiple-joint training is approximately 20% (Weisenthal et al., 2014), none of our participants in either group mentioned any severe injury. Finally, our 1xCircuit_HIIT_ and 2x Circuit_HIIT_ interventions both appeared to improve perception of general health.

From a methodological perspective, the web-based Circuit_HIIT_ employed here was not customized to account for individual strengths and weaknesses, which would be desirable in connection with future applications. In addition, although the participants were instructed to perform the Circuit_HIIT_ “all-out,” their heart rates revealed that the intensity of exercise was actually relatively “moderate.” We did not monitor ratings of perceived exertion after the CircuitHIIT, which should be done in future studies of this kind. In addition, we cannot be certain that the participants performed each exercise correctly, which is a general limitation of mobile-based exercise. Moreover, our home workouts were assessed by monitoring heart rate and we cannot entirely exclude the possibility that someone other than the participant him/herself was wearing the sensor. Furthermore, seasonal variation in free-living activity may explain in part the decline in submaximal heart rate from T_0_ to T_1_. In this study the groups were matched according to their VO_2peak_ and not functional strength. Since the performances in functional strength were heterogenous among the participants (as evidence, e.g., by the standard deviation in **Table 5**) this might explain why no differences were detectable between groups. The current investigation was designed to assess the responses of, in particular, untrained individuals to mobile CircuitHIIT and future evaluation of more long-term responses (e.g., >10 weeks) is required.

## Conclusion

Four weeks of either one or two 6-min micro-sessions of multi-stimulating, circuit-like, multiple-joint training daily improves certain parameters of functional strength and certain dimensions of quality of life in untrained individuals. However, such exercise programs do not enhance cardio-respiratory fitness, having no effect in particular on peak oxygen uptake.

## Author Contributions

All authors listed have made a substantial, direct and intellectual contribution to the work, and approved it for publication.

## Conflict of Interest Statement

The authors declare that the research was conducted in the absence of any commercial or financial relationships that could be construed as a potential conflict of interest.
